# miR-145-5p regulates hepatocellular carcinoma malignant advancement and immune escape via downregulation of AcylCoA synthase ACSL4

**DOI:** 10.17305/bb.2024.11209

**Published:** 2024-12-07

**Authors:** Dingxue Wang, Wenqi Huang, Gao Li

**Affiliations:** 1 Oncology Department, The First Affiliated Hospital of Guizhou University of Traditional Chinese Medicine, Guiyang, China

**Keywords:** Hepatocellular carcinoma, HCC, miR-145-5p, ACSL4, proliferation, immune escape

## Abstract

Hepatocellular carcinoma (HCC) exhibits a subtle onset, high incidence rates, and low survival rates, becoming a substantial threat to human health. Hence, it is crucial to discover fresh biomarkers and treatment targets for the early detection and management of HCC. CCK-8, scratch-wound, and transwell assays were used to evaluate the biological properties of HCC cell lines (Huh-7 and Hep3B). Bioinformatics analysis identified the downstream target mRNA of miR-145-5p as acyl-CoA synthetase long-chain family member 4 (ACSL4). RT-qPCR was used to test miR-145-5p and ACSL4 levels. Transwell chambers were used to co-incubate purified CD8^+^ T cells and HCC cells for 48 h, and the effect of CD8^+^ T cells on apoptosis in HCC cells was detected by flow cytometry. A subcutaneous graft tumor model was constructed, and ELISA kits were used to assess cytokine levels and CD8^+^ T cell activation markers. HCC cells showed a decline in miR-145-5p levels and a rise in ACSL4 levels. Overexpression of miR-145-5p hindered HCC cell proliferation, migration, and invasion, while stimulating CD8^+^ T cell activation. Conversely, overexpression of ACSL4 enhanced the malignant biological properties of HCC cells and reduced the effect of CD8^+^ T cells, while silencing ACSL4 had the opposite effect. miR-145-5p targeted and downregulated ACSL4, while overexpression of miR-145-5p weakened the promotion of HCC malignant progression caused by ACSL4 overexpression. Additionally, overexpression of miR-145-5p and silencing ACSL4 were effective in inhibiting tumor growth *in vivo*. In conclusion, miR-145-5p targets and downregulates ACSL4, leading to the inhibition of HCC malignant progression and preventing immune escape in HCC cells.

## Introduction

The incidence of liver cancer in 2020 ranked sixth among global cancer cases, with mortality rising to third, according to statistics [1, 2]. There were approximately 906,000 new liver cancer cases, accounting for 4.7% of all cancer cases during that period, and 830,000 liver cancer-related deaths, representing 8.3% of total cancer-related deaths [3]. Hepatocellular carcinoma (HCC) accounted for 85%–90% of primary liver cancers [4]. Due to the gradual onset and inconspicuous early symptoms of HCC, most individuals are diagnosed at middle or advanced stages, often accompanied by local infiltration or distant metastasis, making surgery unfeasible [5, 6]. Furthermore, most patients experience HCC recurrence within a short period post-surgery, and their five-year survival rate is only 18% [7, 8]. Therefore, elucidating the molecular mechanisms underlying the occurrence and progression of HCC and identifying early diagnostic markers are crucial to reducing HCC recurrence and mortality rates. MicroRNAs (miRNAs), a class of endogenous RNAs with a length of approximately 19–24 nucleotides, play key regulatory roles within cells [9, 10]. Existing literature reports that miR-145-5p functions as a tumor suppressor gene, inhibiting the progression of various cancers, such as prostate, colorectal, and bladder cancers [11–13]. Additionally, one study has shown that miR-145-5p levels are decreased in HCC cells [14]. However, the current state of research on how miR-145-5p impacts HCC development remains insufficient. Previous research has shown that Acyl-CoA synthetase long-chain family member 4 (ACSL4) enhances HCC malignant progression and may serve as a valuable biomarker for the early diagnosis of the disease [15, 16]. Notably, Liao et al. [17] reported that ACSL4 is closely associated with ferroptosis and immune escape in tumors. However, the influence of ACSL4 on immune escape in HCC cells remains unclear. In this study, we measured miR-145-5p levels in HCC and identified ACSL4 as a downstream target gene of miR-145-5p using bioinformatics analysis, followed by functional investigation. Based on these findings, we explored the effects of miR-145-5p and ACSL4 on the malignant progression and immune escape of HCC. This research aims to provide a novel approach for the early diagnosis and targeted treatment of HCC. Table 1 lists the abbreviations used in this paper and their full names.

## Materials and methods

### Clinical tissue samples

Samples of tissues were gathered from 43 individuals diagnosed with HCC at the First Affiliated Hospital of Guizhou University of Traditional Chinese Medicine, including both cancerous and adjacent tissues. All samples were cleaned and preserved in liquid nitrogen. The Ethics Committee at the First Affiliated Hospital of Guizhou University of Traditional Chinese Medicine approved this study, and written consent for sample collection was obtained from all patients.

### Cell culture and treatment

The human liver epithelial cell line (THLE-2) was provided by Pricella Biotechnology Co., Ltd. (Wuhan, Hubei, China). The HCC cell lines Hep3B (SNL-082) and Huh-7 (SNL-085) were obtained from Sunncell Biotechnology Co., Ltd. (Wuhan, Hubei, China). Cells were cultured in DMEM medium (11965092, Gibco, Grand Island, NY, USA) supplemented with 10% fetal bovine serum (A5670701, Gibco) and 1% penicillin/streptomycin antibiotics (15140122, Gibco). The medium was changed every three days, and cells were passaged at a ratio of 1:3. Incubation was carried out at 37 ^∘^C with 5% CO_2_. MiR-145-5p mimics, ACSL4 short hairpin RNA (sh-ACSL4), ACSL4 overexpression plasmids (OE-ACSL4), and negative controls (mimics-NC, sh-NC, and OE-NC) were purchased from Sangon Biotech (Shanghai, China). Using Lipofectamine 3000 (L3000150, Invitrogen, Carlsbad, CA, USA), the plasmids were transfected into HCC cells following the manufacturer’s instructions.

### RT-qPCR

Total RNA was extracted by lysing tissue samples and cultured cells using Trizol reagent (R0016, Beyotime, Shanghai, China). cDNA was synthesized using AMV reverse transcriptase (2621, TAKARA, Tokyo, Japan). Target genes were amplified using TB Green FAST qPCR mix (CN830S, TAKARA) with cDNA as a template. Relative gene expression levels of miR-145-5p and ACSL4 were normalized to U6 and GAPDH, respectively, using the 2^-ΔΔCt^ method.

The primer sequences were as follows: miR-145-5p: F: 5′-GCCGAGGTCCAGTTTTCCCAGGA-3′, R: 5′-AGAACAGTATTTCCAGGAAT-3′; U6: F: 5′-CCCTTCGGGGACATCCGATA-3′, R: 5′-TTTGTGCGTGTCATCCTTGC-3′; ACSL4: F: 5′-GGCACGCGGTTCCTTTTT-3′, R: 5′-AGCCGACAATAAAGTACGCAA-3′; GAPDH: F: 5′-ATGTTGCAACCGGGAAGGAA-3′, R: 5′-CGCCCAATACGACCAAATCAGA-3′.

### CCK-8 assay

Huh-7 and Hep3B cells were seeded into 96-well plates at a density of 2.0×10^ImEquation2^ cells/well. Once cells adhered to the well walls, the original medium was replaced with 200 µL of fresh medium. CCK-8 reagent (C0038, Beyotime) was added (20 µL/well) at 24, 48, 72, and 96 h. After a 2-h incubation, OD450 values were measured using a microplate reader.

### Scratch-wound assay

Huh-7 and Hep3B cells were trypsin-digested, and 1 mL of cell suspension (3.0 × 10^ImEquation3^ cells/mL) was aspirated using a sterile pipette tip and inoculated into 6-well plates with pre-drawn horizontal lines. Once the cells were completely attached to the surface and reached over 80% confluency, the culture medium was aspirated and discarded. A 20-µL pipette tip was used to create a perpendicular scratch on the bottom of the 6-well plate. Detached cells at the scratch site were removed by washing with PBS, and the wells were filled with serum-free medium. The progression of scratch closure (cell migration) was observed using an inverted fluorescence microscope (DM IL LED, Leica, Heidelberg, Germany) at 0 and 48 h. Scratch widths were measured using ImageJ software (version 1.54h, Wayne Rasband, National Institute of Mental Health, USA) to evaluate the cell migration rate.

### Transwell assay

The Matrigel matrix gel (354230, Corning, Tewksbury, MA, USA) was placed in the freezer compartment of the refrigerator to melt and then removed. The matrix gel was diluted with serum-free medium, and 100 µL of the diluted gel was added to each Transwell (Corning), followed by overnight incubation. A total of 200 µL of Huh-7 and Hep3B cell suspension (1.0 × 10^ImEquation4^ cells/mL) was seeded into the upper chamber, while the lower chamber was filled with complete medium. After 24 h of incubation, cells in the upper chamber were cleared using cotton swabs. The remaining cells were fixed with 4% paraformaldehyde (P0099, Beyotime) and stained with crystal violet solution (C0121, Beyotime). Fields of view were randomly selected and captured using an inverted fluorescence microscope to count the number of invasive cells.

### Isolation of CD8^**+**^ T cells

Venous blood (10 mL) was collected from fasting healthy blood donors, diluted with PBS, and layered over Ficoll separation solution (4550-OP, Sigma-Aldrich, St. Louis, MO, USA). After centrifugation at low speed, the white membrane layer at the interface between plasma and the stratified liquid, representing peripheral blood mononuclear cells (PBMCs), was carefully aspirated. CD8^+^ T cells were isolated from PBMCs using the CD8^+^ T cell sorting kit (11348D, Invitrogen). PBMCs (1.0 × 10^ImEquation5^) were centrifuged, resuspended in PBS buffer, and mixed with a biotin-labeled antibody, followed by incubation at 4 ^∘^C for 5 min. PBS and CD8^+^ T cell microsphere antibody were then added, and the mixture was incubated for 10 min. The separation column was pre-soaked with PBS, and the prepared cell suspension was applied to it. The unlabeled cells that flowed through the separation column were identified as CD8^+^ T cells.

### Flow cytometry

CD8^+^ T cells were co-incubated with HCC cells at a 15:1 ratio (CD8^+^ T cells: tumor targeT cells) for 48 h (CD8^+^ T cells in the upper chamber, HCC cells in the lower chamber) using Transwell chambers and 24-well plates. After the 48-h co-culture, HCC cells were collected and gently mixed with 500 µL of Binding Buffer. Next, 5 µL of propidium iodide (PI, DA0021, Solarbio, Beijing, China) was added and incubated for 15 min in the dark. To assess CD8^+^ T cell cytotoxicity, samples were transferred to tubes, and flow cytometry (BD FACSCalibur™ , BD Biosciences, San Jose, CA, USA) was used to detect apoptosis in HCC cells. CD8^+^ T cells were collected after co-culture with HCC cells, washed twice with PBS, and resuspended in Binding Buffer. Following this, PI and Annexin-V-FITC (C1062S, Beyotime) were added and incubated for 15 min to detect apoptosis in CD8^+^ T cells.

### CD8^**+**^ T cell markers assay

CD8^+^ T cells were collected after co-culture, centrifuged, and resuspended in sterile PBS (1.0 × 10^ImEquation6^/mL). PE-labeled tumor necrosis factor-α (TNF-α) antibody (12-7349-41, Invitrogen) and Granzyme B antibody (12-8899-41, Invitrogen) were added, along with FITC-labeled interferon-γ (IFN-γ) antibody (53-7319-42, Invitrogen) and Perforin antibody (11-9994-42, Invitrogen). The mixture was incubated in the dark for 30 min. Samples were then analyzed using flow cytometry, and experimental data were processed and quantified with FlowJo software (v10.8, BD Biosciences).

### Bioinformatics analysis

The target mRNA of miR-145-5p was identified using the Home-miRWalk (http://mirwalk.umm.uni-heidelberg.de/), GEPIA (http://gepia.cancer-pku.cn/), TargetScanHuman 8.0 (https://www.targetscan.org/vert/_80/), and miRDB (https://mirdb.org/) databases. The results from all four databases were intersected, and ACSL4 was identified as the target gene.

### Dual-luciferase reporter assay

As previously described [18], the wild-type (WT) and mutant (MUT) nucleotide sequences of the 3′UTR of ACSL4 were amplified by PCR using human genomic DNA as a template and then cloned into the pmirGLO vector (Promega, WI, USA) to construct luciferase reporter vectors. Using Lipofectamine 3000, combinations of WT and mimics-NC, WT and mimics, MUT and mimics-NC, as well as MUT and mimics were transfected into Huh-7 and Hep3B cells. After 48 h of incubation, luciferase activity in HCC cells was evaluated using the Dual-Lucy Assay Kit (D0010, Solarbio).

### Western blot

RIPA lysis buffer (P0013B, Beyotime) was used to lyse cells and extract proteins, while the BCA kit (P0012, Beyotime) was used to assess protein concentrations. After gel electrophoresis, the samples were transferred onto PVDF membranes (Invitrogen) and blocked for 1 h. The membranes were washed and incubated overnight at 4 ^∘^C with ACSL4 primary antibody (PA5-27137, 1:1000, Invitrogen), Perforin primary antibody (ab16074, 1:1500, Abcam, Cambridge, MA, USA), or Granzyme B primary antibody (MA1-80734, 1:1000, Invitrogen). The following day, after three washes, the membranes were incubated with a secondary goat anti-rabbit IgG antibody (31460, 1:10,000, Invitrogen) for 2 h. ECL chemiluminescent reagent (34577, Invitrogen) was evenly applied to the membranes and visualized using a gel imaging system (iBright CL1500, Invitrogen). Grayscale values were analyzed using ImageJ software, with GAPDH (MA1-16757, 1:1000, Invitrogen) as the internal reference.

### Subcutaneous tumor model

Healthy BALB/c mice were obtained from Vitalriver (Beijing, China) and acclimatized for one week at a constant temperature of 22 ^∘^C. Each group consisted of six mice, randomly divided into four groups. Each nude mouse received a subcutaneous injection of 200 µL of Huh-7 cell suspension (5 × 10^ImEquation7^ cells per mouse) transfected with sh-NC, sh-ASCL4, mimics-NC, or miR-145-5p mimics in the right axilla. Tumor size was measured weekly using vernier calipers to calculate the tumor volume. On day 28, blood samples were collected via eyeball blood extraction. Mice were then anesthetized and euthanized, and tumors were excised completely, weighed, and photographed to record the tumor mass. This experiment was approved by the Experimental Animal Ethics Committee of the First Affiliated Hospital of Guizhou University of Traditional Chinese Medicine.

### Immunohistochemistry

After fixation with 4% paraformaldehyde, tumor tissues were routinely dehydrated, embedded in paraffin, and sectioned at a thickness of 4–5 µm. The sections were deparaffinized using xylene (247642, Sigma-Aldrich) and subjected to microwave-based antigen retrieval. The tissue sections were treated with 3% H_2_O_2_ solution for 25 min to block endogenous peroxidase activity. Subsequently, the sections were evenly covered with drops of 5% bovine serum albumin (BSA, ST023, Beyotime) and incubated for 30 min to block non-specific binding. The Ki67 antibody (PA5-114437, 1:1000, Invitrogen) was applied to the sections and incubated at 37 ^∘^C for 90 min. The samples were then incubated with an HRP-labeled secondary antibody (1:10,000) for 20 min. DAB (DA1010, Solarbio) was used for color development, and the reaction was terminated with tap water. The sections were counterstained with Mayer’s Hematoxylin (MHS16, Sigma-Aldrich), sealed with neutral gum, and observed under a microscope.

### Immunofluorescence

The tissue sections were permeabilized by applying 0.3% Triton X-100 (X100, Sigma-Aldrich) for 15 min and then blocked with BSA for 2 h. The sections were incubated overnight at 4 ^∘^C with CD4 antibody (41-0042-82, 1:100, Invitrogen) and CD8 antibody (13-0081-82, 1:200, Invitrogen). The following day, the sections were incubated with FITC-labeled secondary antibody (1:10,000) in the dark for 1 h. Finally, the sections were stained with DAPI staining solution (C1005, Beyotime) for 10 min and observed using a fluorescence microscope. Fluorescence intensity was quantified by processing the images with ImageJ software.

**Figure 1.**
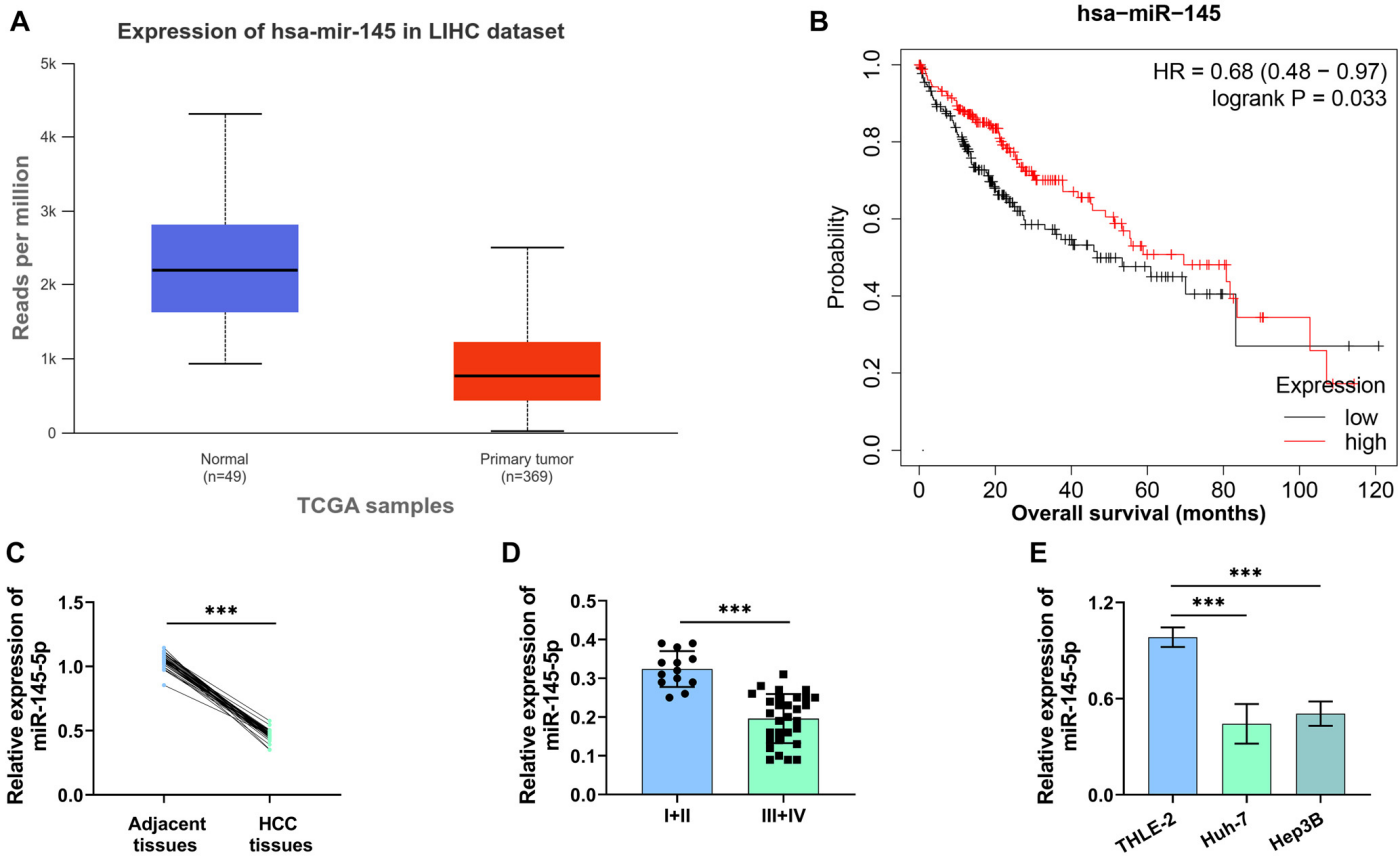
**MiR-145-5p level in HCC cells.** (A) MiR-145-5p levels in HCC tissue (G1, *n* ═ 372) and normal tissue (Normal, *n* ═ 50) samples were analyzed using the UALCAN database; (B) The link between miR-145-5p levels and survival rates of individuals with HCC was examined using the Kaplan–Meier plotter website; (C and D) RT-qPCR detected miR-145-5p levels in different tissues (*n* ═ 43) and assessed miR-145-5p levels in stage I+II (*n* ═ 13) and stage III+IV (*n* ═ 30); (E) RT-qPCR assessed miR-145-5p levels in normal liver cells (THLE-2) and HCC cells (Huh-7 and Hep3B). ***P < 0.001. HCC: Hepatocellular carcinoma.

## ELISA

The collected mouse blood was left to stand for 1 h at room temperature. Once the blood coagulated naturally and serum separated, the supernatant was collected after centrifugation. The secretion levels of IFN-γ and TNF-α in CD8^+^ T cells, as well as IFN-γ, TNF-α, Perforin, and Granzyme B levels in serum, were quantified by ELISA. Human IFN-γ (PI511), human TNF-α (PT518), mouse IFN-γ (PI508), and mouse TNF-α (PT512) kits were obtained from Beyotime. Mouse Perforin ELISA kit (D721128) and Granzyme B ELISA kit (D721076) were sourced from Sangon Biotech. The ELISA well plate was filled with cell culture supernatant or serum and incubated for 2 h. After two washes with PBS, the corresponding antibody was added and incubated for 1 h. Next, HRP-labeled Streptavidin was added and incubated in the dark for 20 min, followed by the addition of TMB solution for 30 min. Finally, the termination solution was added and thoroughly mixed, and the concentration was determined by measuring the OD_450_ value.

### Data processing and analysis

All experiments were performed with a minimum of three repetitions, and results are reported as the mean ± standard deviation. Statistical analysis and image plotting were conducted using SPSS 26.0 software (IBM SPSS Statistics 26) and GraphPad Prism 9.0. The Student’s *t*-test was used to evaluate differences between two groups, while analysis of variance (ANOVA) was applied to compare multiple groups. *P < 0.05, **P < 0.01, ***P < 0.001 denotes that there were a large difference.

### Ethical statement

This study was approved by the Ethics Committee of the First Affiliated Hospital of Guizhou University of Traditional Chinese Medicine (Approval No. YK-20210702).

## Results

### MiR-145-5p level in HCC cells

The levels of miR-145-5p in HCC tissues (G1, *n* ═ 372) and normal tissues (Normal, *n* ═ 50) were analyzed using the UALCAN database (https://ualcan.path.uab.edu/). The results demonstrated that miR-145-5p was significantly downregulated in HCC tissues compared to normal tissues (Figure 1A). Kaplan–Meier survival analysis (http://kmplot.com/analysis/) revealed that patients with low miR-145-5p expression exhibited a poorer survival rate compared to those with high miR-145-5p expression (Figure 1B). RT-qPCR analysis further confirmed that miR-145-5p levels were significantly reduced in HCC tissues and cells compared to normal tissues and cells (Figure 1C and 1E). Additionally, miR-145-5p expression in tumor tissues of HCC patients in stages III+IV (*n* ═ 30) was lower than in those at stages I+II (*n* ═ 13), indicating that miR-145-5p expression is associated with tumor stage (Figure 1D).

**Figure 2.**
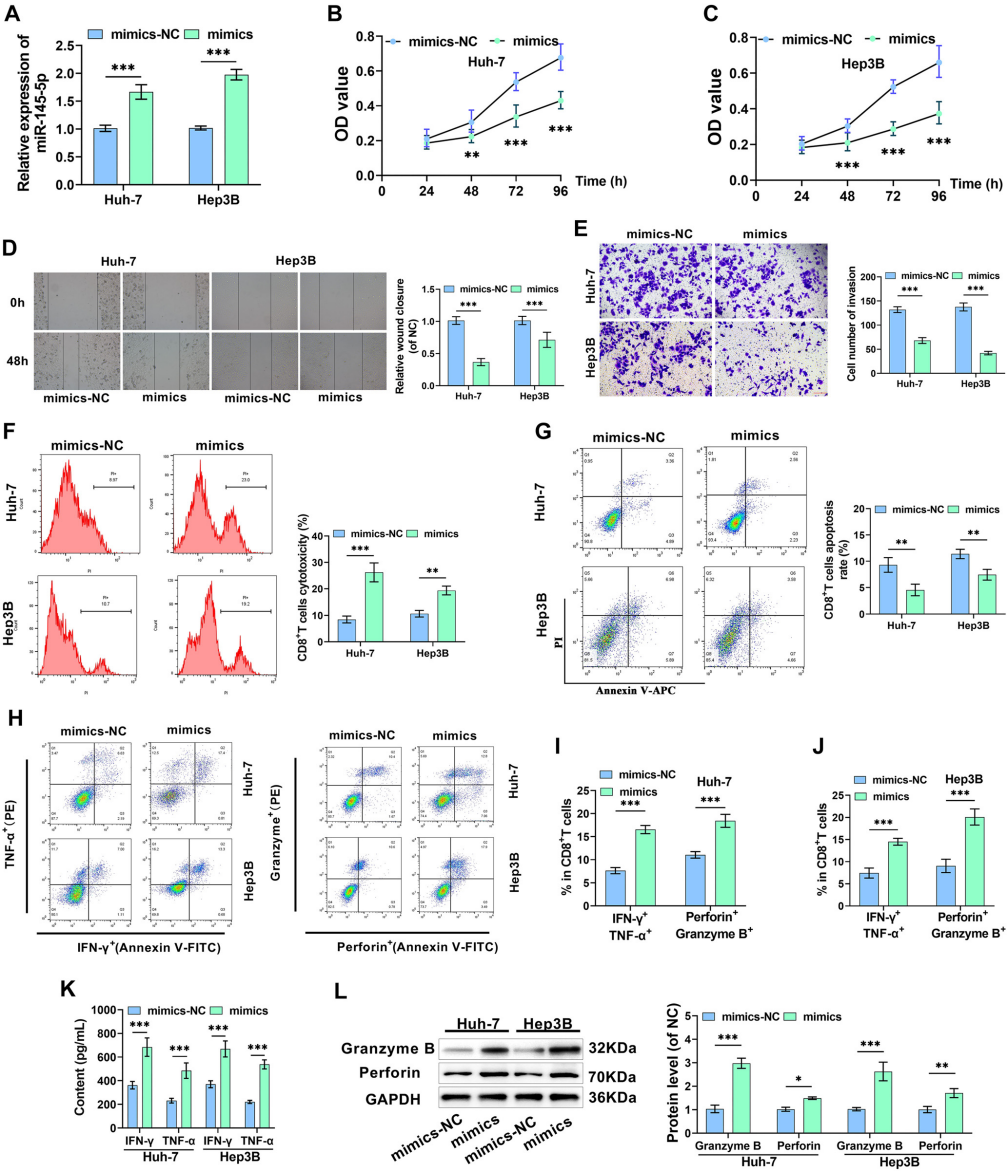
**Overexpression of miR-145-5p suppresses malignant biological behavior and immune escape in HCC cells.** (A) RT-qPCR assessed miR-145-5p levels in differenT cells; (B and C) CCK-8 assay determined the proliferation state of cells after miR-145-5p overexpression; (D) Cell migration was examined via the scratch-wound assay; (E) The number of invasive cells was quantified using the Transwell assay; (F and G) Flow cytometry measured the cytotoxicity and apoptosis of CD8^+^ T cells; (H–J) Flow cytometry detected levels of CD8^+^ T cell activation markers; (K) Cytokine levels of IFN-γ and TNF-α in supernatants were assessed using ELISA kits; (L) Perforin and Granzyme B levels were examined via Western blot. *P < 0.05, **P < 0.01, ***P < 0.001. HCC: Hepatocellular carcinoma; IFN-γ: Interferon-γ.

**Figure 3.**
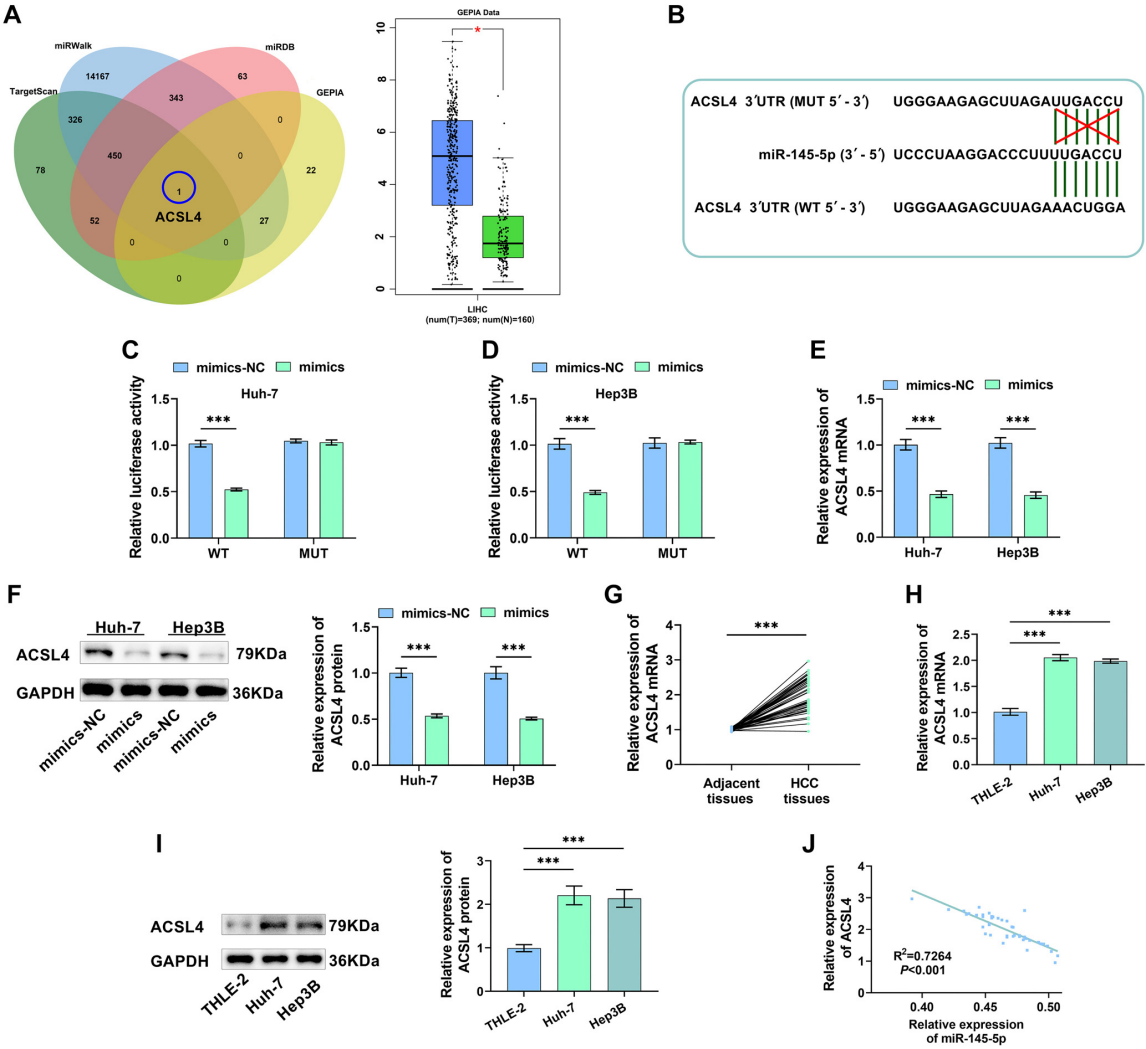
**Screening and verification of miR-145-5p target genes.** (A) Screening for ACSL4 and assessing ACSL4 levels; (B) The sequences showing miR-145-5p and ACSL4 binding were predicted by TargetScan; (C and D) Dual-Luciferase reporter assays verified that miR-145-5p targets and regulates ACSL4 expression; (E and F) ACSL4 levels in differenT cells were examined after miR-145-5p overexpression; (G and H) ACSL4 mRNA levels were assessed by RT-qPCR; (I) ACSL4 protein levels were examined using Western blot in HCC cells; (J) Pearson correlation analysis assessed the correlation between ACSL4 and miR-145-5p expression levels in HCC tissues (*n* ═ 43). ***P < 0.001. ACSL4: Acyl-CoA synthetase long-chain family member 4.

### Overexpression miR-145-5p suppresses malignant biological behavior and immune escape in HCC cells

Huh-7 and Hep3B cells exhibited a marked increase in miR-145-5p levels after being transfected with mimics, which fulfilled the requirements of subsequent experiments (Figure 2A). CCK-8 assay findings indicated a marked decline in cell proliferation in Huh-7 and Hep3B cells due to overexpression of miR-145-5p (Figure 2B and 2C). The scratch-wound assay revealed a notable decrease in cell migration ability in cells overexpressing miR-145-5p (Figure 2D). The Transwell assay implied that cell invasion capabilities were weakened following overexpression of miR-145-5p (Figure 2E). To study the influence of miR-145-5p levels on CD8^+^ T cell function, we incubated CD8^+^ T cells with HCC cells for 48 h. Flow cytometry data demonstrated a significant enhancement in the cytotoxicity of CD8^+^ T cells toward Huh-7 and Hep3B cells after miR-145-5p was overexpressed. The CD8^+^ T cell apoptosis rate was significantly reduced, and the quantity of IFN-γ, TNF-α, Perforin, and Granzyme B-positive cells was markedly increased (Figure 2F–2J). In addition, ELISA findings indicated a notable increase in IFN-γ and TNF-α levels in co-culture supernatants due to overexpression of miR-145-5p (Figure 2K). By Western blot, Perforin and Granzyme B levels, protein markers of CD8^+^ T cell activation, were markedly increased following overexpression of miR-145-5p (Figure 2L). In conclusion, overexpression of miR-145-5p hindered the malignant biological behaviors of HCC cells and boosted the killing impact of CD8^+^ T cells on HCC cells.

**Figure 4.**
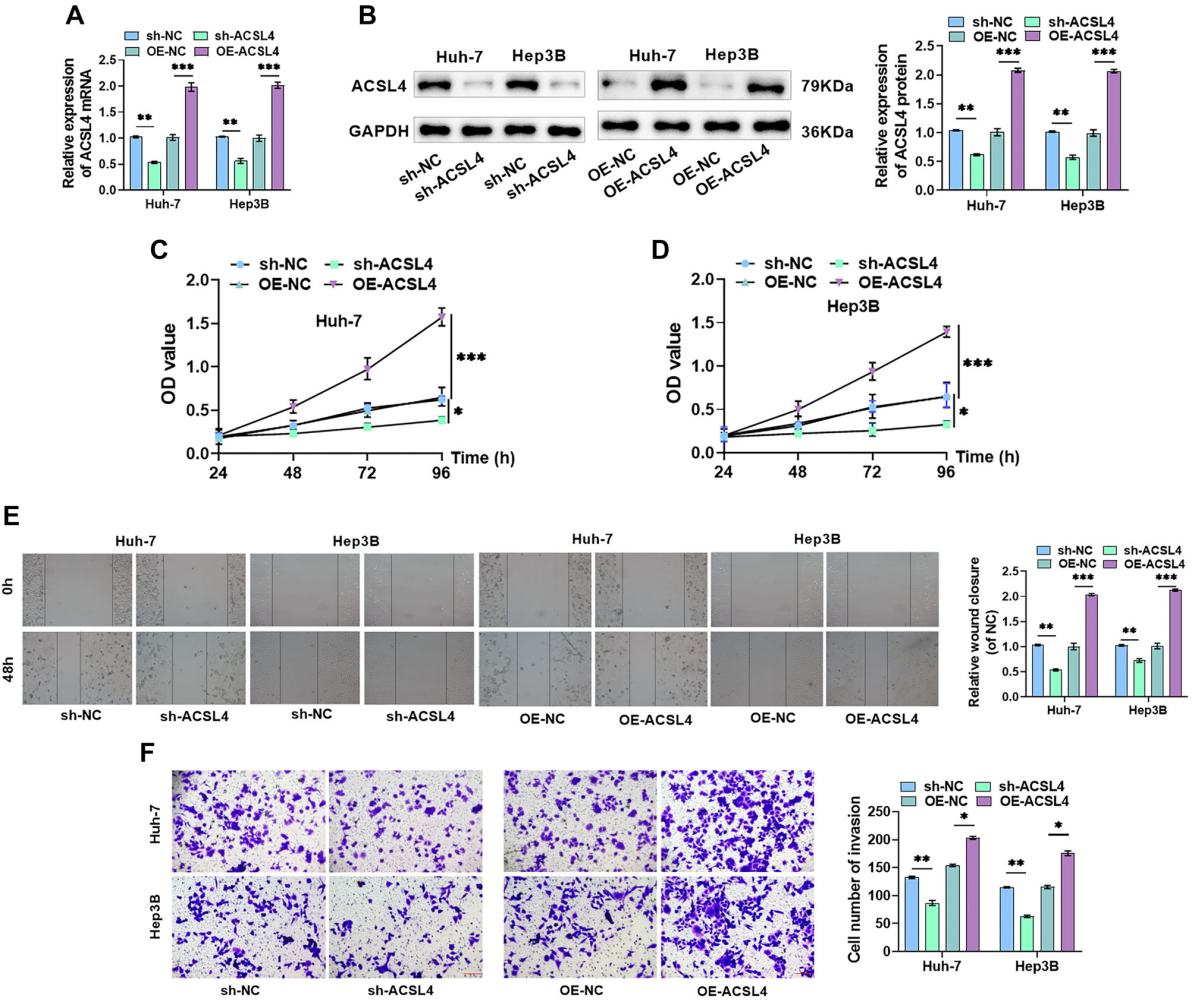
**ACSL4 promotes malignant biological behavior in HCC cells.** (A and B) HCC cells were transfected with sh-ACSL4 or OE-ACSL4, and RT-qPCR and Western blot detected ACSL4 mRNA and protein levels; (C and D) CCK-8 assay determined cell proliferation; (E) Cell migration was examined via the scratch-wound assay; (F) The number of invasive cells was quantified using the Transwell assay. *P < 0.05, **P < 0.01, ***P < 0.001. HCC: Hepatocellular carcinoma; ACSL4: Acyl-CoA synthetase long-chain family member 4.

### Screening of the genes targeted by MiR-145-5p

The downstream target genes of miR-145-5p were predicted using the GEPIA, TargetScanHuman, miRDB, and Home-miRWalk databases, and their intersection identified the ACSL4 gene. According to the GEPIA database, there is a notable increase in ACSL4 levels in HCC (Figure 3A). Figure 3B displays the ACSL4-WT nucleotide sequences and the ACSL4-MUT nucleotide sequences. To further explore this interaction, the target binding of miR-145-5p to ACSL4 was verified via the Dual-Luciferase reporter assay. The findings revealed that overexpression of miR-145-5p markedly reduced the luciferase signal in the WT group, while no significant influence was observed in the MUT group, further confirming that ACSL4 is a downstream gene regulated by miR-145-5p (Figure 3C and 3D). Additionally, overexpression of miR-145-5p decreased ACSL4 levels, further validating the targeted and negative regulatory effect of miR-145-5p on ACSL4 expression (Figure 3E and 3F). ACSL4 exhibited high expression levels in HCC, consistent with GEPIA database findings (Figure 3G–3I). The relative expression levels of ACSL4 mRNA and miR-145-5p in HCC tissues (*n* ═ 43) were detected by RT-qPCR, followed by Pearson correlation analysis of their expression levels. The results indicated that ACSL4 expression was negatively correlated with miR-145-5p levels (Figure 3J).

### ACSL4 promotes malignant biological behavior in HCC cells

To investigate the influence of ACSL4 on HCC malignant progression, we transfected sh-ACSL4 or OE-ACSL4 into Huh-7 and Hep3B cells and verified transfection efficiency. Transfection with sh-ACSL4 resulted in a significant decline in ACSL4 expression, while ACSL4 levels were markedly elevated after transfection with OE-ACSL4 (Figure 4A and 4B). As revealed by the CCK-8 assay, silencing ACSL4 significantly inhibited the proliferation of Huh-7 and Hep3B cells, while overexpression of ACSL4 significantly enhanced cell proliferation (Figure 4C and 4D). Furthermore, cell migration and invasion were significantly reduced following silencing of ACSL4, whereas overexpression of ACSL4 had the opposite effect (Figure 4E and 4F). These results indicate that silencing ACSL4 inhibits malignant biological behavior, while overexpression of ACSL4 promotes the malignant characteristics of HCC cells.

### ACSL4 promotes immune escape in HCC cells

**Figure 5.**
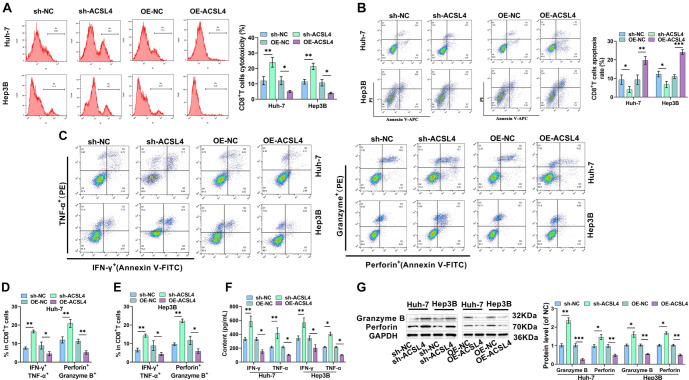
**ACSL4 promotes immune escape in HCC cells.** (A) Flow cytometry measured the cytotoxicity of CD8^+^ T cells; (B) CD8^+^ T cell apoptosis rates were assessed using flow cytometry; (C–E) Flow cytometry detected levels of CD8^+^ T cell activation markers; (F) Cytokine levels of IFN-γ and TNF-α in supernatants were assessed using ELISA kits; (G) Perforin and Granzyme B levels were examined via Western blot. *P < 0.05, **P < 0.01, ***P < 0.001. ACSL4: Acyl-CoA synthetase long-chain family member 4; HCC: Hepatocellular carcinoma; IFN-γ: Interferon-γ.

The toxicity of CD8^+^ T cells toward HCC cells increased notably after silencing ACSL4, while the apoptosis rate of CD8^+^ T cells was significantly reduced. In contrast, overexpression of ACSL4 significantly decreased the toxicity of CD8^+^ T cells and notably increased their apoptosis rate (Figure 5A and 5B). Silencing ACSL4 led to a significantly higher number of IFN-γ, TNF-α, Perforin, and Granzyme B-positive cells, while overexpression of ACSL4 produced the opposite effect (Figure 5C–5E). Additionally, silencing ACSL4 resulted in notably higher levels of IFN-γ and TNF-α (Figure 5F) and significantly elevated expression levels of Perforin and Granzyme B proteins (Figure 5G). These findings align with the flow cytometry results and suggest that silencing ACSL4 enhances the killing impact of CD8^+^ T cells on HCC cells. In contrast, overexpression of ACSL4 led to a marked reduction in IFN-γ and TNF-α levels, and Perforin and Granzyme B proteins were also downregulated, indicating that overexpression of ACSL4 weakened the killing effect of CD8^+^ T cells.

### miR-145-5p partially reverses the impact of ACSL4 in HCC cells

**Figure 6.**
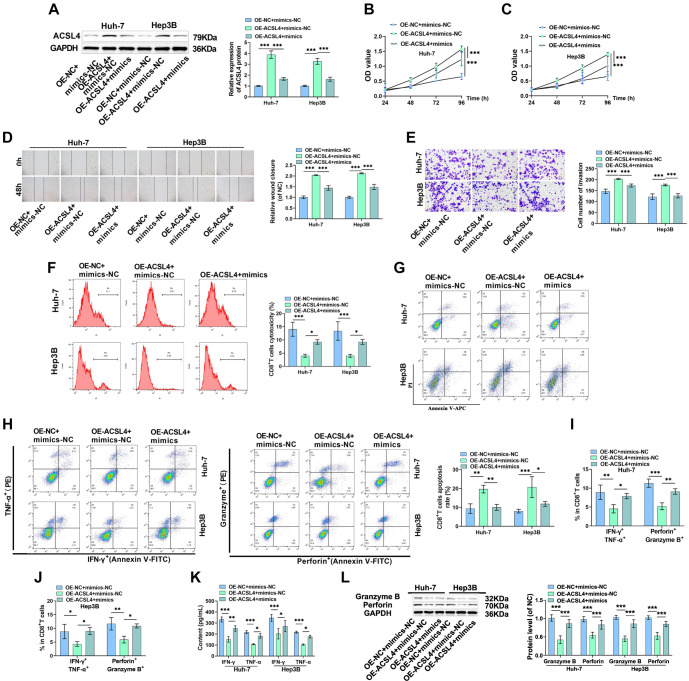
**miR-145-5p reverses ACSL4-induced promotion of HCC cell proliferation and immune escape.** (A) ACSL4 levels were examined using Western blot; (B and C) CCK-8 assay determined cell proliferation; (D) Cell migration was examined using the scratch-wound assay; (E) The number of invasive cells was quantified using the Transwell assay; (F and G) Flow cytometry measured the cytotoxicity and apoptosis of CD8^+^ T cells; (H–J) Flow cytometry detected levels of CD8^+^ T cell activation markers; (K) IFN-γ and TNF-α levels in supernatants were assessed using ELISA kits; (L) Perforin and Granzyme B levels in CD8^+^ T cells were examined via Western blot. *P < 0.05, **P < 0.01, ***P < 0.001. ACSL4: Acyl-CoA synthetase long-chain family member 4; HCC: Hepatocellular carcinoma; IFN-γ: Interferon-γ.

Next, we investigated whether miR-145-5p targets ACSL4 to inhibit malignant biological behavior and immune escape in HCC cells. Transfection with OE-ACSL4 significantly increased ACSL4 levels, whereas co-transfection with OE-ACSL4 and miR-145-5p mimics partially reduced ACSL4 levels, indicating that overexpression of miR-145-5p downregulated ACSL4 expression (Figure 6A). Cell proliferation was markedly enhanced by overexpression of ACSL4, but overexpression of miR-145-5p attenuated the proliferative effect caused by ACSL4 overexpression (Figure 6B and 6C). Similarly, ACSL4 overexpression increased the migratory and invasive potential of HCC cells, but this effect was diminished by overexpression of miR-145-5p (Figure 6D and 6E). Overexpression of ACSL4 significantly reduced the toxicity of CD8^+^ T cells while increasing their apoptosis rate and decreasing the number of IFN-γ, TNF-α, Perforin, and Granzyme B-positive cells. This indicates that ACSL4 overexpression inhibited CD8^+^ T cell activation. However, overexpression of miR-145-5p mitigated these effects (Figure 6F–6J). Furthermore, ACSL4 overexpression led to a marked reduction in IFN-γ and TNF-α levels (Figure 6K) and a significant decline in Perforin and Granzyme B levels (Figure 6L). These effects were partially reversed by overexpression of miR-145-5p. In summary, overexpression of ACSL4 promotes malignant biological behavior and immune evasion in HCC cells. In contrast, overexpression of miR-145-5p attenuates the effects of ACSL4 overexpression, suggesting that miR-145-5p hinders the malignant biological behavior and immune escape of HCC cells by targeting ACSL4.

### Silencing ACSL4 or overexpression miR-145-5p suppresses tumor growth and immune escape *in vivo*

**Figure 7.**
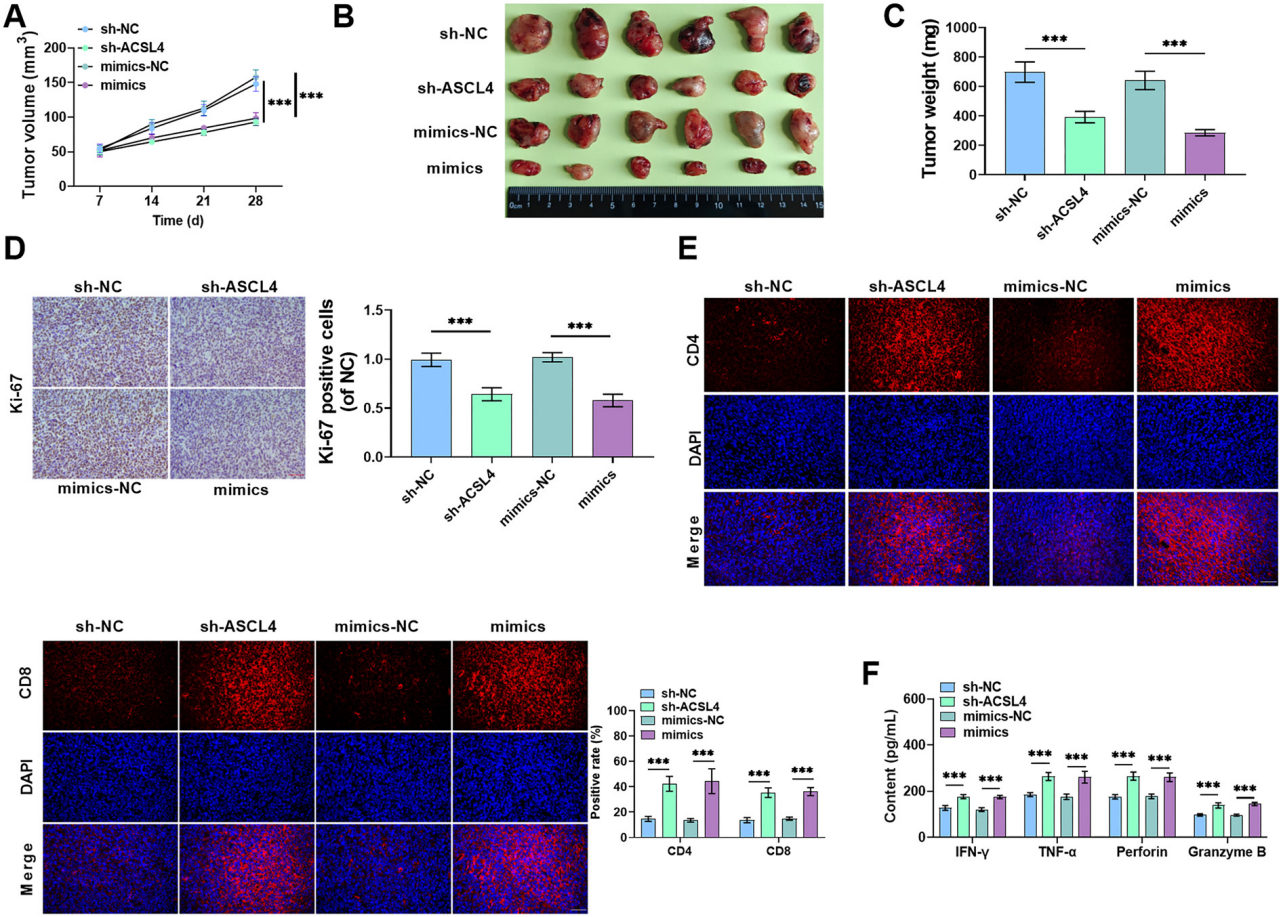
**Silencing ACSL4 or overexpression of miR-145-5p reduces tumor growth and immune escape *in vivo*.** (A) Huh-7 cell suspensions were injected subcutaneously into nude mice. The size of subcutaneous tumors was measured using a vernier caliper on days 7, 14, 21, and 28 (*n* ═ 6); (B) On day 28, nude mice were anesthetized and sacrificed, followed by tumor removal and imaging; (C) Tumor weights were recorded; (D) Immunohistochemistry assessed Ki-67 expression in tumor tissues; (E) Immunofluorescence detected CD4 and CD8 expression in tumor tissues; (F) IFN-γ, TNF-α, Perforin, and Granzyme B levels in serum were measured using ELISA kits. ***P < 0.001. IFN-γ: Interferon-γ; ACSL4: Acyl-CoA synthetase long-chain family member 4.

Finally, to investigate the effects of ACSL4 and miR-145-5p on tumor growth *in vivo*, we constructed a subcutaneous transplantation tumor model. Overexpression of miR-145-5p or silencing of ACSL4 notably inhibited tumor growth in terms of volume and weight (Figure 7A–7C). Immunohistochemistry analysis revealed that Ki-67 positivity was markedly reduced in tumor tissues following either ACSL4 silencing or miR-145-5p overexpression (Figure 7D). Immunofluorescence results demonstrated a significant increase in CD4 and CD8 protein positivity after silencing ACSL4 or overexpressing miR-145-5p, confirming the infiltration of CD8^+^ T cells into HCC tissues (Figure 7E). Moreover, silencing ACSL4 or overexpressing miR-145-5p significantly increased the levels of IFN-γ, TNF-α, Perforin, and Granzyme B in the blood of mice (Figure 7F). These findings suggest that silencing ACSL4 or overexpressing miR-145-5p inhibits HCC malignant progression and prevents tumor cells from undergoing immune escape.

## Discussion

Recently, the role of miRNAs in promoting or inhibiting various malignant tumor biological behaviors has become a hot topic in the study of tumor pathogenesis [19, 20]. MiR-145-5p is a microRNA linked to numerous cancer types [21]. Ji et al. [22] discovered that overexpression of miR-145-5p could weaken the growth of prostate cancer cells. Fan et al. [23] demonstrated that miR-145-5p can inhibit the malignant progression of esophageal cancer by modulating the ABRACL level in a targeted manner. We analyzed miR-145-5p levels in different tissue samples through the UALCAN database and discovered a marked decline in miR-145-5p in HCC cells compared to normal cells, which matched the RT-qPCR findings. Additionally, according to the Kaplan–Meier plotter platform, HCC patients with low levels of miR-145-5p exhibited lower survival rates than those with high expression levels, which aligned with the findings of Wang et al. [24]. Previous research has demonstrated a strong connection between tumor development and the tumor microenvironment. The tumor microenvironment is constituted by stromal cells (including interstitial cells, endothelial cells, and epithelial cells) and immune cells infiltrated around the tumor [25, 26]. As a category of small molecular proteins secreted by immune cells and other cell types, cytokines include lymphokines and interferons and play a crucial role in immune system reactions [27, 28]. CD8^+^ T lymphocytes represent a critical subset of T cells that release cytotoxic molecules like Perforin and Granzyme B. They also secrete a variety of cytotoxic factors in the vicinity of targeT cells, promoting immune effector functions [29, 30]. IFN-γ, as a crucial mediator in cellular immune reactions, not only enhances immune regulation but also exhibits antiviral and anticancer properties [31, 32]. TNF-α, an inflammatory cytokine, is released by CD8^+^ T cells and modulates the immune response [33, 34]. We found that overexpression of miR-145-5p markedly hindered the malignant biological properties of HCC cells. Notably, overexpression of miR-145-5p enhanced IFN-γ and TNF-α levels and increased the expression of Perforin and Granzyme B, implying that miR-145-5p can improve CD8^+^ T cell activation. This result indicated that overexpressing miR-145-5p hindered the malignant advancement and immune escape of HCC. To further investigate how miR-145-5p suppresses HCC malignant advancement, we identified the predicted target gene ACSL4 for miR-145-5p using different databases. Furthermore, a Dual-Luciferase reporter assay provided additional confirmation that miR-145-5p can indeed target and negatively regulate ACSL4. As observed, ACSL4 levels were high in HCC tissues but low in normal tissues, aligning with the results documented by Sun et al. [35]. ACSL4 is predominantly found in the endoplasmic reticulum, mitochondria, and peroxisomes and can mediate cellular ferroptosis [36]. Notably, it has been shown that blocking the transcription of ACSL4 can effectively inhibit the growth of liver cancer, suggesting that ACSL4 may act as a pro-oncogene [37]. Additionally, Liao et al. [17] reported that ACSL4 is involved in CD8^+^ T cell-mediated antitumor immunity.

**Table 1:** The list of abbreviations

HCC	Hepatocellular carcinoma
ACSL4	Acyl-CoA synthetase long-chain family member 4
CCK-8	Cell counting kit-8
miRNAs	MicroRNAs
ACS	Acyl-CoA synthetases
sh-ACSL4	ACSL4 short hairpin RNA
OE-ACSL4	ACSL4 overexpression plasmids
PBMCs	Peripheral blood mononuclear cells
TNF-α	Tumor necrosis factor-α
IFN-γ	Interferon-γ
PI	Propidium iodide
WT	Wild-type
MUT	Mutant-type
BSA	Bovine serum albumin

In this research, silencing ACSL4 was observed to suppress HCC cell migration, invasion, and proliferation, while promoting CD8^+^ T cell activation, thereby hindering immune escape in HCC cells. In contrast, overexpression of ACSL4 facilitated HCC malignant progression and immune escape. Nevertheless, overexpression of miR-145-5p weakened the impact of ACSL4 overexpression in promoting HCC malignant progression and immune escape. This result further confirms that miR-145-5p mediated ACSL4 levels to inhibit HCC malignant progression. In addition, we constructed a mouse subcutaneous transplantation tumor model and found that overexpression of miR-145-5p and silencing of ACSL4 both promoted CD8^+^ T cell infiltration in HCC and inhibited malignant progression and immune escape in HCC.

## Conclusion

To sum up, there was a reduction in miR-145-5p levels in HCC tissues and cells. However, it negatively regulated ACSL4 levels in a targeted manner, inhibiting HCC cell migration, invasion, and proliferation, while promoting CD8^+^ T cell activation, thereby alleviating the malignant progression and immune escape of HCC. MiR-145-5p and ACSL4 hold promise as biomarkers and novel therapeutic targets for HCC. There are still some shortcomings in this research, and the signaling pathways through which miR-145-5p/ACSL4 regulate tumor advancement can be further explored in future studies. Furthermore, future studies should also elucidate how ACSL4 impacts CD8^+^ T cell activity within tumors.

## Supplemental data

**Highlights:**

1. miR-145-5p level in HCC cells is declined.

2. Over-expression miR-145-5p suppresses the malignant advancement of HCC cells.

3. miR-145-5p negatively regulated the ACSL4 expression level.

4. Over-expression ACSL4 promoted HCC malignant advancement and inhibited CD8^+^ T cell activation.

5. Over-expression miR-145-5p reversed such phenomena as HCC cell proliferation and immune escape promoted by overexpression ACSL4.

**Graphical abstract.**
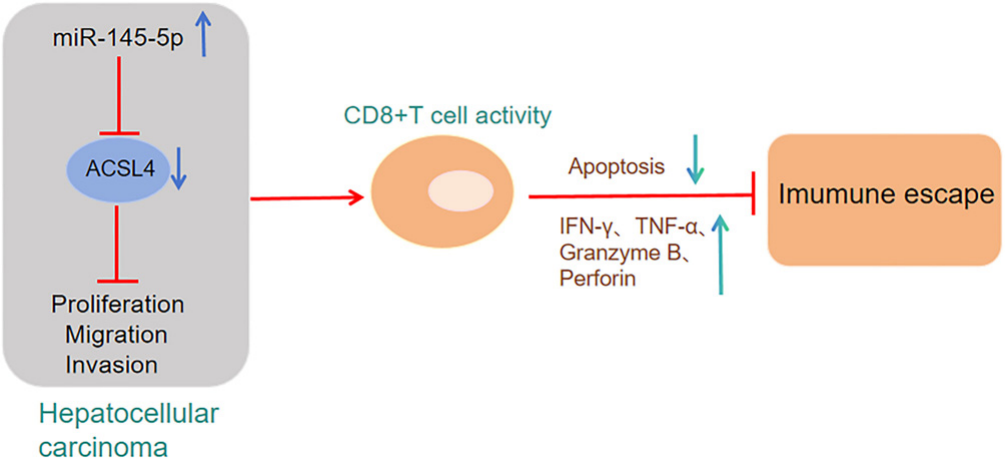
miR-145-5p targets and downregulates ACSL4, activates CD8^+^ T cells, which in turn suppresses the malignant progression and immune escape of HCC cells.

## Data Availability

The data that support the findings of this study are available from the corresponding author, upon reasonable request.
